# Gene Expression Profiling of Adipose Tissue in Enshi Black Pigs Subjected to Cold Stress

**DOI:** 10.3390/vetsci13050442

**Published:** 2026-04-30

**Authors:** Tong Zhang, Liang Wang, Shuo Yang, Guangdong Hu, Dongjie Zhang

**Affiliations:** 1 College of Animal Science and Technology, Shihezi University, Shihezi 832000, China; zhangtong20152045@163.com; 2 Institute of Animal Husbandry, Heilongjiang Academy of Agricultural Sciences, Harbin 150086, China; wlwl448@163.com (L.W.); hqyangshuo@foxmail.com (S.Y.)

**Keywords:** adipose, cold stress, Enshi black pigs, KEGG, metabolism

## Abstract

To investigate the response mechanism of cold-resistant Enshi black pig breeds under cold stress, nine Enshi black pigs were randomly divided into three groups with three pigs in each: a control group (18 ± 2 °C for 58 d), a cold-stress-acclimated group (3 to 8 °C to −17 to −21 °C for 58 d), and an acute cold stress group (−17 to −21 °C for 3 d). RNA-seq technology was used to analyze mRNA and lncRNA expression patterns in subcutaneous adipose tissue under cold stress. The results showed that, under acute cold stress, many metabolic pathways were activated, including those involved in rapid energy supply (e.g., the citric acid cycle/TCA cycle, fatty acid degradation and metabolism, and glycolysis/gluconeogenesis), signal transduction pathways (e.g., PI3K Akt, MAPK, PPAR, HIF-1, mTOR, and FoxO), and immune and cellular homeostasis pathways (chemokine signaling pathway, T cell receptor signaling, Toll-like receptor signaling, and apoptosis and autophagy regulation). Under cold stress acclimation, metabolic regulatory pathways (e.g., AMPK, mTOR, FoxO, HIF-1, glycolysis/gluconeogenesis, and fatty acid degradation), immune and inflammatory regulatory pathways (Toll-like receptors, NOD like receptors, and T/B cell receptor signaling pathways), and signal transduction and cell homeostasis pathways (MAPK, PI3K Akt, NF-κB, Notch signaling pathways, apoptosis, and autophagy regulation) were continuously activated to ensure the stability of adipose tissue structure and function. Acute cold stress activated more pathways than cold stress acclimation, but both led to significant changes in energy metabolism. The results identified the molecular regulatory mechanisms of adipose tissue under cold stress, providing a basis for the subsequent breeding of new cold-resistant pig breeds.

## Simple Summary

Cold stress is a common challenge in pig farming. Newborn piglets are particularly sensitive to cold due to a lack of adipose tissue, especially brown adipose fat. Cold stress can lead to hypothermia, weakened immunity, diarrhea, and even death in piglets, causing significant economic losses to the swine industry. Exploring the energy remodeling and steady-state maintenance mechanisms of cold-resistant pig breeds under cold stress, as well as the regulatory factors and signaling pathways related to cold stress, can provide a basis for the development of cold prevention and control measures, optimization of feed formulas, and cultivation of cold-resistant varieties in the pig industry, thereby reducing economic losses.

## 1. Introduction

Adipose tissue is primarily composed of mature adipocytes, preadipocytes, and immune cells and innervated by blood vessels and nerves. Adipose tissue is no longer considered a passive energy storage organ but rather the largest endocrine and immune organ in the body that is capable of secreting various bioactive substances. It plays a significant role in resisting cold through non-shivering thermogenesis. Adipose tissue comprises both brown adipose tissue (BAT) and white adipose tissue (WAT). BAT is a thermogenic tissue; under cold stress, cells in WAT function similarly to those in BAT (i.e., beige fat), a phenomenon known as white fat beigeing [1]. Promoting BAT thermogenesis and WAT beigeing can increase energy expenditure and reduce fat deposition. BAT thermogenesis primarily relies on the UCP1 thermogenic pathway [2]. In addition, there are UCP1-independent thermogenic pathways, including Ca^2+^-ATPase 2b calcium cycling and the futile creatine cycle [3,4].

Pigs are born lacking BAT. One hypothesis suggests that this is due to mutations in the UCP1 gene [5]. Another study suggests that during the early embryonic development of pigs, the absence of EBF2 expression prevents mesodermal precursor cells from differentiating into brown fat cells [6]. Because pigs are born lacking BAT, it remains unclear how they utilize adipose tissue to generate heat under cold stress. Reports indicate that in cold environments, cold-resistant breeds such as Tibetan and Min pigs exhibit significantly higher expression of UCP3. This factor can function similarly to UCP1, and thus, white fat undergoes beigeing [7].

Long non-coding RNAs (lncRNAs) are non-coding RNA molecules with a length exceeding 200 nucleotides. LncRNAs are involved in regulating the remodeling of energy metabolism in adipose tissue under cold induction. For example, LncRNA lnc266 can adsorb miR-16-1-3p, eliminate its inhibition of UCP1 expression, promote the differentiation of preadipocytes into brown adipocytes, and stimulate the expression of thermogenic genes [8]. LncRNA2310069B03Rik can inhibit UCP1 activity under cold induction [9]. Our research group also discovered an lncRNA, lncRNA44154, previously shown to be induced by cold stress, that plays a regulatory role in the proliferation and differentiation of adipocytes [10].

Enshi black pigs are an indigenous black pig breed of China that have demonstrated good fat storage ability and adaptation to cold or wet conditions [11]. Compared with commercial pig breeds, Enshi black pigs are better suited for identifying key genes involved in temperature regulation, because they are well-known for their adaptability to a mountainous environment and cold–wet tolerance [12]. In this study, they were used as experimental animals to analyze the mRNA and lncRNA expression profiles of subcutaneous adipose tissue on their backs after varying degrees of cold stress using RNA-seq, and to explore the possible regulatory mechanisms of lncRNAs in this process. This study aims to reveal the thermogenic mechanisms underlying cold resistance in pigs.

## 2. Materials and Methods

### 2.1. Experimental Grouping and Sample Collection

**Figure 1:**
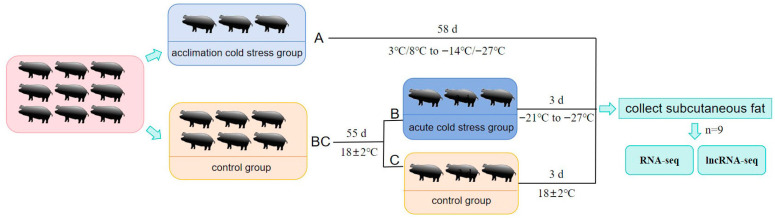
Experimental design of the cold stress model for Enshi black pigs.

Nine healthy Enshi black sows, aged 3 months and weighing 14 ± 2 kg, were provided by Huazhong Agricultural University. The nine sows were randomly divided into three groups: a control group (group C), a cold-stress-acclimated group (group A), and an acute cold stress group (group B), with three pigs in each group. Groups C and B were kept in a normal shed with the temperature controlled at 18 ± 2 °C. Group A was kept in an outdoor semi-open shed for 55 days, during which the outdoor temperature ranged from 3 to 8 °C to −17 to −21 °C. When the outdoor temperature reached −17 to −21 °C, group B was transferred to an outdoor semi-open shed for 3 days (Figure 1 and Figure S1). Subsequently, all experimental pigs were slaughtered, and the subcutaneous adipose tissue harvested from the dorsal region was immediately stored in liquid nitrogen for subsequent experimental analysis and use.

### 2.2. Extraction and Quality Assessment of Total RNA

Total RNA from adipose tissue was extracted using the TRIzol reagent (Carlsbad, CA, USA). Weigh 50 mg tissue sample and dissolve it in 1 mL of Trizol reagent. Incubate the mixture at room temperature (15–30 °C) for 5 min. Add 0.2 mL chloroform, vortex vigorously for 15 s, and incubate for 3 min. Centrifuge the mixture at 12,000× *g* (4 °C) for 15 min. After centrifuging, aspirate the upper aqueous phase and transfer it to a new EP tube. Add 0.5 mL of isopropanol to the collected aqueous phase, and incubate for 10 min. Then, centrifuge at 12,000× *g* (4 °C) for 10 min. Discard the supernatant, add 1 mL of 75% ethanol, and centrifuge at 7500× *g* (4 °C) for 5 min. Discard the supernatant again, and air-dry the RNA precipitate until no visible ethanol residue remains. Dissolve the RNA precipitate in an appropriate volume of RNase-free water. The purity and concentration of RNA were assessed using a NanoDrop 2000 spectrophotometer (Thermo Fisher Scientific, Waltham, MA, USA). The OD260/OD280 ratio was required to be between 1.8 and 2.4, and the concentration was required to be at least 50 ng/μL. RNA integrity was evaluated using a Bioanalyzer 2100 (Thermo Fisher Scientific, Waltham, MA, USA). The results were judged based on RIN value (≥7), peak shape, tailing, and baseline.

### 2.3. High-Throughput Sequencing and Data Quality Control

The extracted total RNA from the adipose tissue samples was sent to Novogene Biotech Co., Ltd. (Beijing, China). for sequencing on an Illumina NovaSeq 6000 (San Diego, CA, USA) in PE150 mode. After sequencing, the raw data were obtained from the machine, and the clean data were obtained after quality control. Quality control involved removing sequencing fragments containing adapters and low-quality fragments (those with a proportion of N bases greater than 10% and fragments where Q ≤ 10 bases accounted for more than 50% of the entire sequencing fragment). All subsequent analyses were based on the clean data.

### 2.4. Alignment with the Reference Genome

The clean data were aligned to the pig reference genome (*Sus Scrofa* 11.1) using HISAT2 v2.0.5 (https://daehwankimlab.github.io/hisat2/, (accessed on 11 April 2016)) to obtain the positioning information of the sequenced fragments. The aligned sequencing fragments were then assembled using StringTie v1.3.3b (https://ccb.jhu.edu/software/stringtie/, (accessed on 15 February 2017)) to reconstruct the transcriptome for subsequent analysis. Generate a PCA scatter plot to assess sample clustering patterns.

### 2.5. Identification of lncRNAs

LncRNAs were identified from the assembled transcripts. These transcripts were removed, including lowly expressed transcripts with FPKM < 0.5, short transcripts < 200 bp, and those with <2 exons. The coding potential of lncRNAs was analyzed using CNCI, Pfam, and CPC2.

### 2.6. Analysis of DEmRNA and DElncRNAs

Fragments Per Kilobase of transcript sequence per Million mapped reads (FPKM) was used as a measure of gene expression. Genes with |log_2_ (Fold Change)| > 0.58 and *p* < 0.05 were considered differentially expressed genes (DEGs). Target gene prediction for lncRNAs was performed in two ways. Genes located within 10/100 kb of the lncRNA were selected as potential cis-targeted genes. Pearson correlations between genes and lncRNAs were calculated and analyzed to identify trans-targeted genes, with a correlation threshold of ≥0.9. GO and KEGG enrichment analyses of target genes of DElncRNAs were implemented by the clusterProfiler R-3.2.4 (https://www.bioconductor.org/packages/release/bioc/html/clusterProfiler.html, (accessed on 14 October 2015)), in which gene length bias was corrected. The enrichment was considered to be significant when the *p*-value was less than 0.05.

### 2.7. RT-qPCR Validation and Statistical Analysis

RT-qPCR was used to validate the sequencing results. Eight genes and eight lncRNAs were chosen at random, and β-actin was used as a housekeeping gene. The primer sequences used for RT-qPCR are listed in Table S1. PCR reaction system: 2×SYBR Green qPCR Master Mix 10 μL, upstream primer and downstream primer each 0.8 μL, cDNA template (500 ng/μL) 1 μL, ddH_2_O 7.4 μL. PCR reaction procedure: 95 °C 30 s, 95 °C 5 s, and 60 °C 30 s for 40 cycles, 95 °C 15 s, 60 °C 1 min. qPCRsoft 4.0 was used to calculate the Ct value. The 2^−∆∆CT^ method was used to quantify the relative expression levels. A *t*-test was performed using GraphPad Prism 10.5.0 (https://graphpad-prism.cn/, (accessed on 30 May 2025)) to analyze the significant differences in target gene expression between the experimental group and the control group.

## 3. Results

### 3.1. Screening and Analysis of DEGs in the Subcutaneous Adipose Tissue of Enshi Black Pigs After Cold Treatment

The raw data from nine sequencing libraries were purified, yielding approximately 12.66 Gb of clean bases (Table S2).

**Figure 2:**
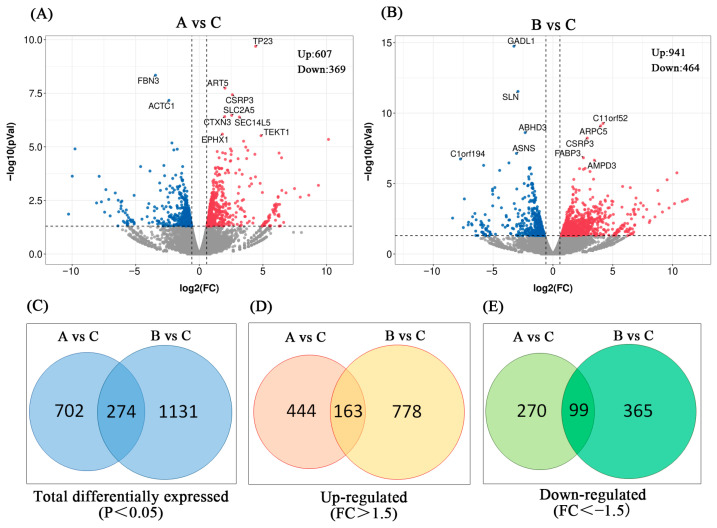
DEGs in the subcutaneous adipose of Enshi black pigs. (**A**) Volcano plot comparing groups A and C (|log_2_FC| > 0.58 and *p* < 0.05). (**B**) Volcano plot comparing groups B and C. (**C**) Total DEGs in the comparison of groups A and B. (**D**) Upregulated DEGs in the comparison of groups A and B. (**E**) Downregulated DEGs in the comparison of groups A and B.

Of the clean reads, 81.54% were uniquely mapped to the reference genome (*Sus scrofa* 11.1) and spliced into putative transcripts (Table S3). The PCA results indicated that individuals within the same group were largely clustered together (Figure S2). However, there were also certain variations within the group. Volcano plots provide an overview of the DEGs between group A vs. group C and between group B vs. group C (Figure 2A,B), where red and blue indicate upregulated and downregulated genes, respectively, and gray indicates genes that were not significantly differentially expressed. According to the above criteria, there were 607 upregulated and 369 downregulated genes in group A, and 941 upregulated and 464 downregulated genes in group B. The top 10 annotated genes exhibiting the greatest fold change are presented in Table 1. Overall, 274 DEGs were simultaneously expressed in these two groups; 702 DEGs were specifically expressed in group A, and 1131 DEGs were specifically expressed in group B (Tables S4 and S5). More genes were activated or inhibited under acute cold stress (Figure 2C–E).

### 3.2. DEGs Were Primarily Enriched in Energy Metabolic Pathways

GO analyses of the DEGs of groups A and B were carried out. The top 20 enriched GO terms of each group are shown in Figure S3A,B. In group A, genes involved in the extracellular matrix were significantly enriched. Under biological processes, oxidoreductase, NADH dehydrogenase, and transmembrane transporter activities were significantly enriched. The DEGs of group B were associated with transporter, acyl-CoA dehydrogenase, and transmembrane transporter activities.

The top 20 KEGG pathways related to DEGs in groups A and B are listed in Figure S3C,D. Other enriched pathways are listed in Tables S6 and S7. Analysis revealed that energy metabolism, including sugar, fatty acid, and amino acid metabolism, was altered under cold treatment. This indicates that oxidative stress, nerve damage, and an enhanced immune response may occur. Metabolic pathways, the PI3K-Akt signaling pathway, fatty acid metabolism, and fatty acid degradation were all significantly enriched in groups A and B.

### 3.3. LncRNAs Were Differentially Expressed in the Subcutaneous Adipose Tissue of Enshi Black Pigs Subjected to Cold Stress

**Figure 3:**
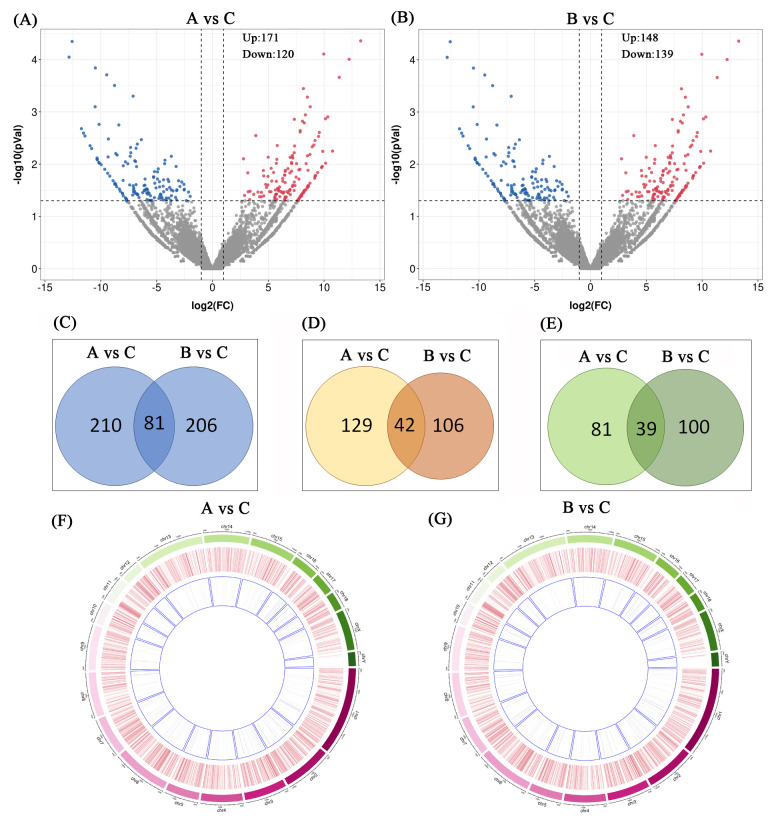
DElncRNAs in the subcutaneous adipose tissue of Enshi black pigs under cold treatment. (**A**) Volcano plot comparing groups A and C. (**B**) Volcano plot comparing groups B and C. (**C**) Total DElncRNAs in the comparison of groups A and B. (**D**) Upregulated DElncRNAs in groups A and B. (**E**) Downregulated DElncRNAs in groups A and B. (**F**) The distribution of DElncRNAs on pig chromosomes for group A. (**G**) The distribution of DElncRNAs on pig chromosomes for group B.

lncRNAs are generally less evolutionarily conserved and more tissue-specific than mRNAs; however, their roles in subcutaneous adipose physiology under cold stress are unclear. Significantly differentially expressed lncRNAs (DElncRNAs) in groups A and B (|log_2_FC| > 0.58 and *p* < 0.05) are listed in Tables S8 and S9. There were 291 DElncRNAs in group A and 287 DElncRNAs in group B (Figure 3A,B). In the figure, red and blue indicate upregulated and downregulated lncRNAs, respectively, and gray indicates that the lncRNAs were not significantly differentially expressed. Across the two cold stress modes, 81 lncRNAs showed the same trend: 42 were upregulated, and 39 were downregulated (Figure 3C–E). The number of upregulated lncRNAs was greater than the number of downregulated lncRNAs and was similar to the results for mRNA. The difference in the number of DElncRNAs between the two groups was not significant. The lncRNAs were widely distributed across all euchromosomes and sex chromosomes (Figure 3F,G). The outermost circle represents the chromosome length of the pig genome, the middle circle represents all lncRNAs detected in this experiment, and the innermost circle represents the DElncRNAs.

### 3.4. The Functional Prediction of DElncRNA in Group A

The target genes of the DElncRNAs were predicted to analyze their functions. In group A, 291 DElncRNAs had 2608 target genes, of which 1943 were cis-targeted, and 665 were trans-targeted. GO analysis indicated that the most enriched GO terms among cis-targeted genes were cytokine receptor binding, defense response, and receptor binding (Figure S4A). The most enriched GO terms of trans-targeted genes were ATP binding, adenyl nucleotide binding, and cytokine activity (Figure S4B).

KEGG pathway analysis indicated that the cis-targeted genes were involved in 75 pathways (Table S10). The trans-targeted genes were involved in 166 pathways (Table S11). Figure S4C,D list the top 20 pathways (according to the criterion of a q-value < 0.05). The pathways were enriched in signal transduction, such as the Jak-STAT signaling pathway, the VEGF signaling pathway, the PI3K-Akt signaling pathway, the cGMP-PKG signaling pathway, cytokine–cytokine receptor interaction, the mTOR signaling pathway, the MAPK signaling pathway, the cAMP signaling pathway, the FoxO signaling pathway, the Rap1 signaling pathway, and the phosphatidylinositol signaling system. Others were enriched in metabolism categories, including the glycolysis/gluconeogenesis pathway and the oxidative phosphorylation pathway. At the same time, several immune-related pathways had also undergone changes, including the cytosolic DNA-sensing and Toll-like receptor signaling pathways.

### 3.5. The Functional Prediction of DE lncRNAs in Group B

In group B, 287 DElncRNAs had 2807 target genes, 2150 cis-targeted genes, and 657 trans-targeted genes. The most enriched GO terms of the cis-targeted genes were cytokine receptor binding, defense response, and stress response. The most enriched GO terms among trans-targeted genes were small molecule binding, hydrolase activity, and nucleotide binding (Figure S5A,B).

KEGG pathway analysis indicated that the cis-targeted genes were involved in 61 pathways (Table S12), and the trans-targeted genes were involved in 166 pathways (Table S13). The top 20 pathways (with q-values < 0.05) are listed in Figure S5C,D. These pathways were enriched in organismal systems, including the immune and endocrine systems. These included the Fc gamma R-mediated phagocytosis pathway, the T-cell receptor signaling pathway, and the Thyroid hormone signaling pathway. Several others were enriched in signal transduction, including the PI3K-Akt, MAPK, and Jak-STAT signaling pathways. The pathways related to metabolism were enriched in amino acid metabolism (cysteine and methionine metabolism) and carbohydrate metabolism (inositol phosphate metabolism).

### 3.6. Validation of Transcriptome Sequencing

**Figure 4:**
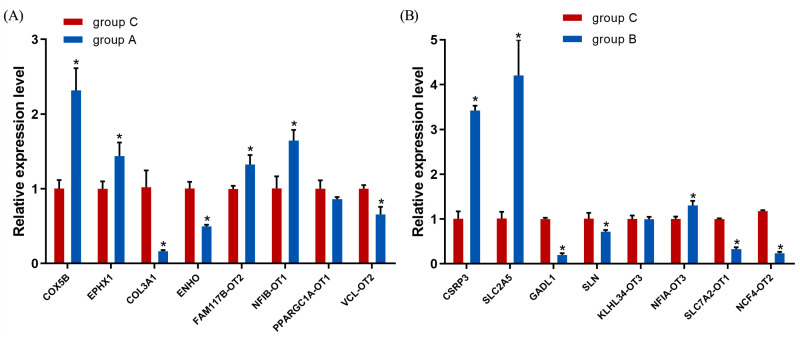
The qRT-PCR results. (**A**) qRT-PCR results for group A. (**B**) qRT-PCR results for group B. The * indicates significant differences (*p *< 0.05).

Eight DEGs and eight DElncRNAs were selected at random to verify the RNA-seq results. The qRT-PCR results showed that the variation trends for genes and lncRNAs were consistent with those from RNA-seq, indicating that the RNA-seq results were reliable (Figure 4).

## 4. Discussion

Cold stress can decrease piglet disease resistance, leading to diarrhea, reduced growth rates, and even death [13]. Moreover, as one of the main heat-producing tissues, adipose tissue responds differently at varying levels of cold stress. Under acute cold stress, β-adrenergic signaling upregulates the expression of genes driving BAT thermogenesis. Long-term cold stress can induce browning of subcutaneous white adipose tissue (scWAT) and increase thermogenesis [14]. However, some studies on mice have shown that under acute cold stress (4 °C for 6 h), if the mice are fasting, WAT will provide fatty acids to BAT to promote the tricarboxylic acid cycle [15]. Currently, research on the mechanism of heat production in adipose tissue under cold stress primarily focuses on BAT [16]. There is limited research on heat production in WAT or adipose tissue of pigs under cold stress. This study characterizes changes in gene expression across coding and non-coding genes in porcine adipose tissue under cold stress, using omics-based analysis of adipose tissue samples from Enshi black pigs, a typical cold-tolerant pig breed. These findings provide preliminary insight into the regulatory mechanisms underlying the maintenance of energy metabolism homeostasis in pigs through adipose metabolism and thermogenesis in response to cold stress.

Due to limited sample availability, this study included only three biological replicates. To mitigate technical variability to the greatest extent possible, the experimental conditions were strictly controlled, coupled with the adoption of a high-sensitivity detection platform. Nevertheless, this study’s findings still have certain inherent limitations. In order to discover more DEmRNA and DElncRNA, the screening parameters were set to |log_2_FC| > 0.58 and *p* < 0.05, but doing so unavoidably increased the number of false positive DEgenes to some extent.

After cold stress, RNA-seq analysis revealed alterations in lipid metabolism-related pathways (including 2-oxocarboxylic acid metabolism, fatty acid metabolism and degradation, and glycerol ester metabolism) in the adipose tissue of Enshi black pigs, as well as in energy-regulation pathways such as AMPK, mTOR, HIF-1, and FoxO. These pathway changes initiate fat mobilization and thermogenesis programs, which convert stored fat into thermogenic substrates and maintain body temperature homeostasis. This is consistent with previous research on Erhualian pigs [17] and Hezuo pigs [18], suggesting that lipid metabolism is a universal adaptation of pig adipose tissue to cold stress. At the same time, changes in adipocyte cytokine signaling and adrenergic signaling pathways enhance adipose tissue’s endocrine regulatory function [19]. The modulation of glutathione metabolism, MAPK, and other pathways can alleviate oxidative damage induced by cold stress in adipocytes and maintain the structural and functional stability of adipose tissue. Cold stress can induce the production of free radicals in animal tissues, thereby activating the antioxidant system to resist oxidative damage, a process confirmed in murine studies [20]. Changes in immune-related pathways, such as chemokine and cytokine receptor interactions, suggest that pig adipose tissue can contribute to the body’s overall defense by activating local immune responses during cold stress [21].

Comparing responses across different levels of cold stress, more pathways were activated under acute cold stress (163 vs. 97). After removing pathways activated under both modes, 74 pathways were activated under acute cold stress, including those related to metabolism, immunity, signal transduction, disease, and cellular physiology. This suggests that under acute strong stimulation, adipose tissue, as an organ involved in metabolism and immune regulation, undergoes stress-induced adaptive activation [22]. There were relatively few biological pathways activated during acclimation to cold stress, with a focus on basal metabolism, signal transduction, and a few disease-related pathways. This suggests that under long-term mild cold stimulation, the stress response of adipose tissue is more inclined toward sustained metabolic adjustment and adaptive regulation than acute, widespread effects [13].

LncRNAs do not encode proteins but rather participate in thermogenesis by regulating target genes [23,24,25]. After Enshi black pigs were subjected to cold stress, a large number of lncRNAs in the adipose tissue underwent changes. The target genes regulated by these lncRNAs involved energy metabolism and substance conversion pathways, signaling pathways, and immune and inflammation-related pathways. By analyzing two levels of cold stress, we found that cold stress signals were transmitted to adipocytes through pathways such as MAPK and PI3K-Akt [26,27], thereby activating energy metabolism pathways, such as fatty acid degradation and glycolysis, to rapidly supply energy and maintain a constant body temperature [28,29]. Simultaneously, immune–inflammatory pathways (the T-cell and Toll-like receptor signaling pathways) were activated [30] to clear damaged cells via apoptosis and autophagy [31], thereby maintaining cell homeostasis.

The biological pathways enriched by the target genes of DElncRNAs in Enshi black pigs subjected to acute cold stress and cold stress acclimation not only share the above characteristics but also exhibit distinct pathways, reflecting differences in the body’s response to varying degrees of cold stress. Under acute cold stress, adipose tissue rapidly breaks down fatty acids, amino acids, and sugars, providing a large amount of substrate and reducing power for immediate heat production. The citrate cycle (the TCA cycle), the PPAR signaling pathway, and fatty acid metabolism were rapidly activated [32,33,34]. Apoptosis is activated to clear damaged cells. Under acclimation to cold stress, there was a shift toward AMPK/cAMP-mediated long-term metabolic regulation [35], adipose tissue remodeling (Notch signaling pathway) [36], gene expression reprogramming (RNA transport and spliceosome) [37,38], and systemic homeostasis adaptation, thereby achieving long-term tolerance to low-temperature environments. These contrasting pathway profiles further reveal that DElncRNAs orchestrate a stage-specific regulatory strategy in response to acute cold stress versus acclimation. Acute stress relies on rapid energy mobilization and clearance of cell damage, while acclimation drives sustained metabolic adaptation, tissue remodeling, and transcriptomic reprogramming. Together, these findings clarify the distinct molecular mechanisms underlying short-term thermogenesis and long-term cold tolerance in Enshi black pigs.

## 5. Conclusions

This study elucidates the response mechanism of subcutaneous adipose tissue in Enshi black pigs, a cold-resistant pig breed, under cold stress. The results showed that cold stress activates pathways associated with energy metabolism, signal transduction, immune function, and metabolic regulation. Specifically, acute cold stress preferentially triggers pathways involved in rapid energy mobilization and thermogenesis, whereas cold acclimation facilitates long-term adaptive metabolic remodeling and alleviates persistent damage to adipose tissue. Future work will focus on in-depth functional characterization of genes and non-coding RNAs that are significantly upregulated under both cold stress modes, with the goal of dissecting their roles in maintaining systemic energy homeostasis under cold stress.

## Figures and Tables

**Table 1:** The top 10 annotated genes exhibiting the greatest fold change in groups A and B.

	Gene	Log_2_ FC	*p* Value	Gene	Log_2_ FC	*p* Value
Group A	ACTL6B	7.48	0.0009	C9orf40	−9.77	1.253 × 10^−5^
	UNC80	6.82	0.0014	TTC36	−7.70	0.0037
	FBXO36	6.63	0.0337	MYL4	−7.12	0.0028
	GSG1L	6.49	0.0011	HEPACAM	−6.61	0.0014
	HOXB9	6.32	0.0049	AMH	−6.40	0.0489
	TULP2	6.26	0.0144	SOSTDC1	−6.38	0.0031
	KISS1	6.20	0.0164	CCL22	−6.17	0.0076
	DRC1	6.19	0.0049	C10orf105	−6.16	0.0038
	SLIT1	6.18	0.0157	HOXB8	−6.09	0.0034
	LMO3	6.08	0.0060	PDX1	−5.87	0.0343
Group B	SPAG5	9.70	0.0005	VEPH1	−8.38	0.0028
	TREH	9.54	5.415 × 10^−6^	Clorf194	−7.72	1.812 × 10^−7^
	KCNN4	8.87	0.0003	TNMD	−7.40	0.0001
	SPP1	8.77	3.093 × 10^−5^	SI	−7.08	0.0086
	ATP6V0D2	7.84	0.0007	CDHR2	−7.05	0.0056
	NEFH	7.55	0.0004	RPH3A	−6.80	0.0071
	PIF1	6.84	0.0085	SYT17	−6.76	0.0087
	LY6G6C	6.81	0.0015	MINAR1	−6.71	0.0023
	KCNIP1	6.80	0.0007	LRRC71	−6.52	0.0013
	UNC80	6.77	0.0010	AMH	−6.44	0.0486

## Data Availability

The original contributions presented in the study are included in the article/Supplementary Materials; further inquiries can be directed to the corresponding authors.

## Supplementary Materials

The following supporting information can be downloaded at https://www.mdpi.com/article/10.3390/vetsci13050442/s1, Figure S1: Temperature changes in the outdoor semi-open shed during the experimental period; Figure S2: GO and KEGG analyses of DEGs. (A) The top 20 GO enrichment analysis terms for the DEGs in group A. (B) The top 20 GO enrichment analysis terms for the DEGs in group B. (C) The top 20 KEGG pathway enrichment terms for the DEGs in group A. (D) The top 20 KEGG pathway enrichment terms for the DEGs in group B; Figure S3: DElncRNAs in the subcutaneous adipose tissue of Enshi black pigs under cold treatment. (A) Volcano plot comparing groups A and C. The red color indicates upregulated lncRNAs, and the blue color indicates downregulated lncRNAs (|log_2_FC| > 0.5 and *P* < 0.05). The gray color indicates the non-statistically significant expressed lncRNAs. (B) Volcano plot of the comparison between groups B and C. (C) Total DElncRNAs in the comparison between groups A and B. (D) Upregulated DElncRNAs in groups A and B. (E) Downregulated DElncRNAs in groups A and B. (F) The distribution of DElncRNAs on pig chromosomes for group A. The outermost circle represents the chromosome length of the pig genome; the middle circle represents all lncRNAs detected in this experiment, and the innermost circle represents the DElncRNAs. (G) The distribution of DElncRNAs on pig chromosomes for group B; Figure S4: Functional prediction of the DElncRNAs of group A. (A) GO analysis of DElncRNAs cis-targeted genes. (B) GO analysis of DElncRNAs trans-targeted genes. (C) Pathway analysis of DElncRNAs cis-targeted genes. (D) Pathway analysis of DElncRNAs trans-targeted genes; Figure S5: Functional prediction of DElncRNAs of group B. (A) GO analysis of DElncRNAs with cis-targeted genes. (B) GO analysis of DElncRNAs with trans-targeted genes. (C) The top 20 pathways of DElncRNAs with cis-targeted genes. (D) The top 20 pathways of DElncRNAs with trans-targeted genes; Table S1: The sequences of primers used for qRT-PCR; Table S2: Sequencing data quality assessment; Table S3: Alignment of all samples with the reference genome; Table S4: DEmRNAs of group A vs. group C; Table S5: DEmRNAs of group B vs. group C; Table S6: KEGG pathway analysis of DEGs of group A vs. group C; Table S7: KEGG pathway analysis of DEGs of group B vs. group C; Table S8: The list of DElncRNAs in group A vs. group C; Table S9: The list of DElncRNAs in group B vs. group C; Table S10: The KEGG pathways of the genes co-located with DElncRNAs in the group acclimated to cold stress; Table S11: The KEGG pathways of the genes co-expressed with DElncRNAs in the group acclimated to cold stress; Table S12: The KEGG pathways of the genes co-located with DElncRNAs in the acute cold stress group; Table S13: The KEGG pathways of the genes co-expressed with DElncRNAs in the acute cold stress group.
