# Supplementary material for: Gene Expression Profiling of Adipose Tissue in Enshi Black Pigs Subjected to Cold Stress
Source: Vet Sci. 2026 Apr 30;13(5):442. doi: 10.3390/vetsci13050442 (PMC13211509; doi:10.3390/vetsci13050442)
Supplement: Supplementary file 1 [file vetsci-13-00442-s001.zip › Fig.S/Fig.S3.pdf]

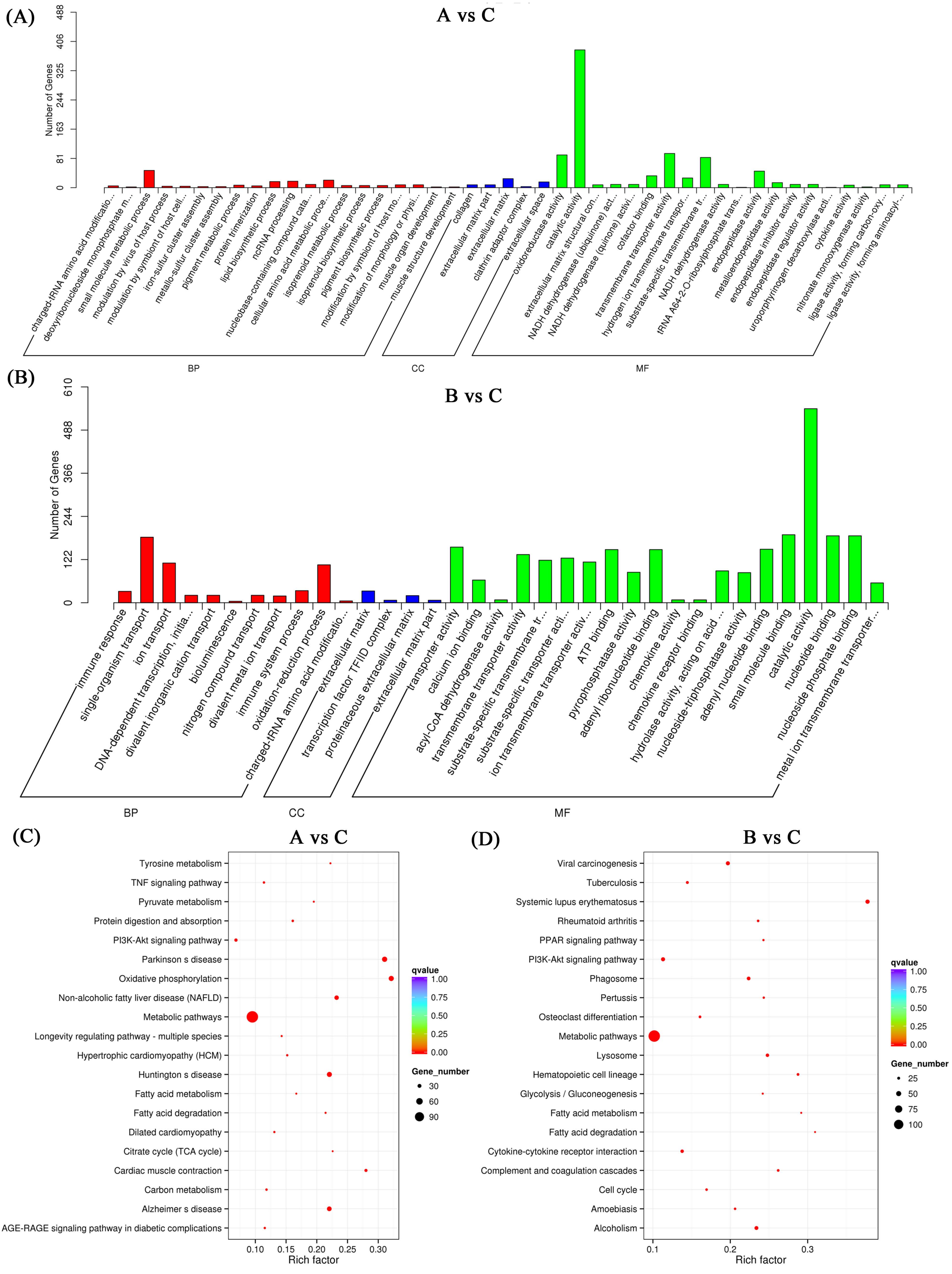

Fig. S3. GO and KEGG analyses of DEGs. (A) The top 20 GO enrichment analysis terms for the DEGs in group A. (B) The top 20 GO enrichment analysis terms for the DEGs in group B. (C) The top 20 KEGG pathway enrichment terms for the DEGs in group A. (D) The top 20 KEGG pathway enrichment terms for the DEGs in group B.
