# Supplementary material for: Gene Expression Profiling of Adipose Tissue in Enshi Black Pigs Subjected to Cold Stress
Source: Vet Sci. 2026 Apr 30;13(5):442. doi: 10.3390/vetsci13050442 (PMC13211509; doi:10.3390/vetsci13050442)
Supplement: Supplementary file 1 [file vetsci-13-00442-s001.zip › Fig.S/Fig.S5.pdf]

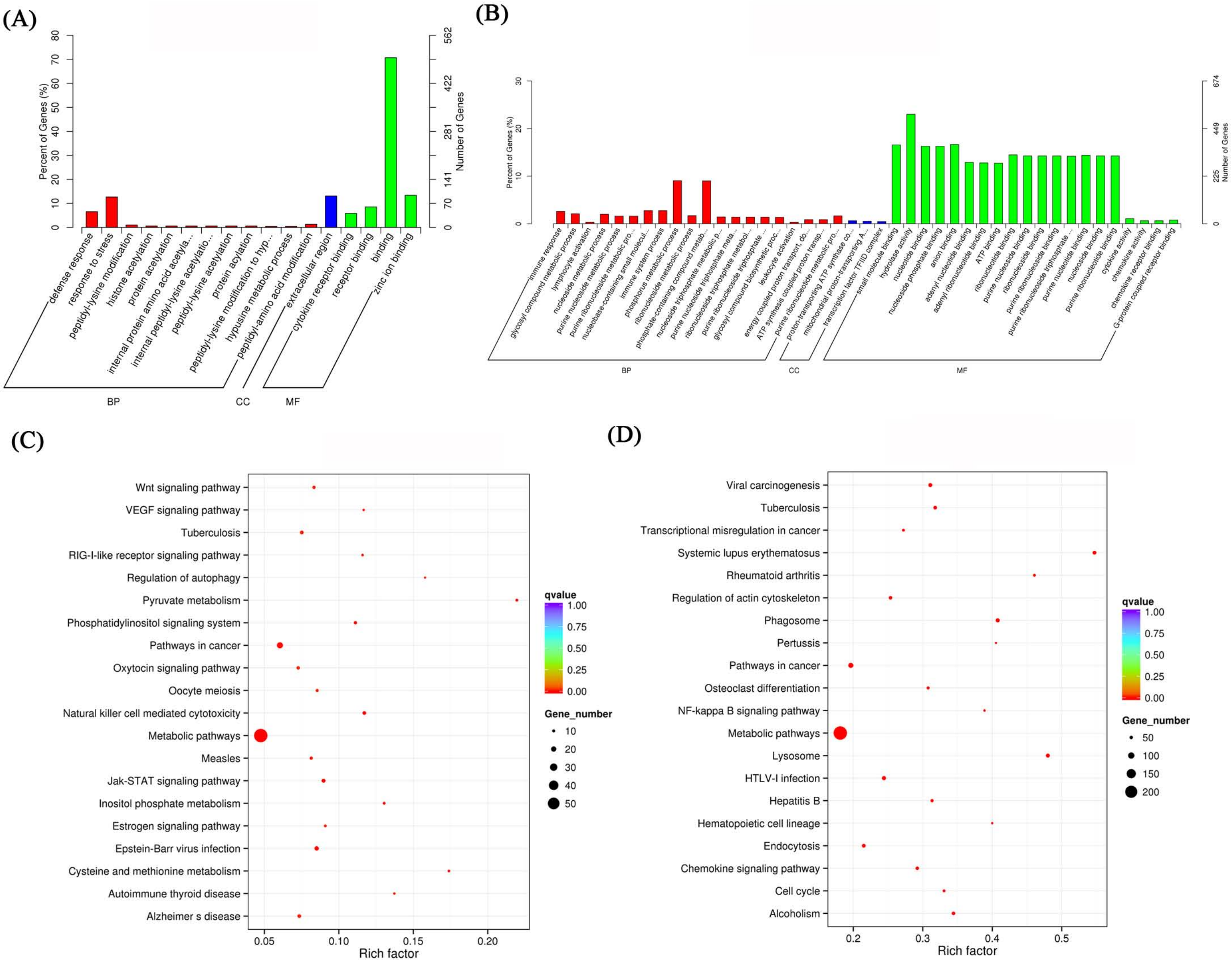

Fig. S5. Functional prediction of DElncRNAs of group B. (A) GO analysis of DElncRNAs with cis-targeted genes. (B) GO analysis of DElncRNAs with trans-targeted genes. (C) The top 20 pathways of DElncRNAs with cis-targeted genes. (D) The top 20 pathways of DElncRNAs with trans-targeted genes.
