# Supplementary figures and images for: Gene Expression Profiling of Adipose Tissue in Enshi Black Pigs Subjected to Cold Stress
Source: Vet Sci. 2026 Apr 30;13(5):442. doi: 10.3390/vetsci13050442 (PMC13211509; doi:10.3390/vetsci13050442)

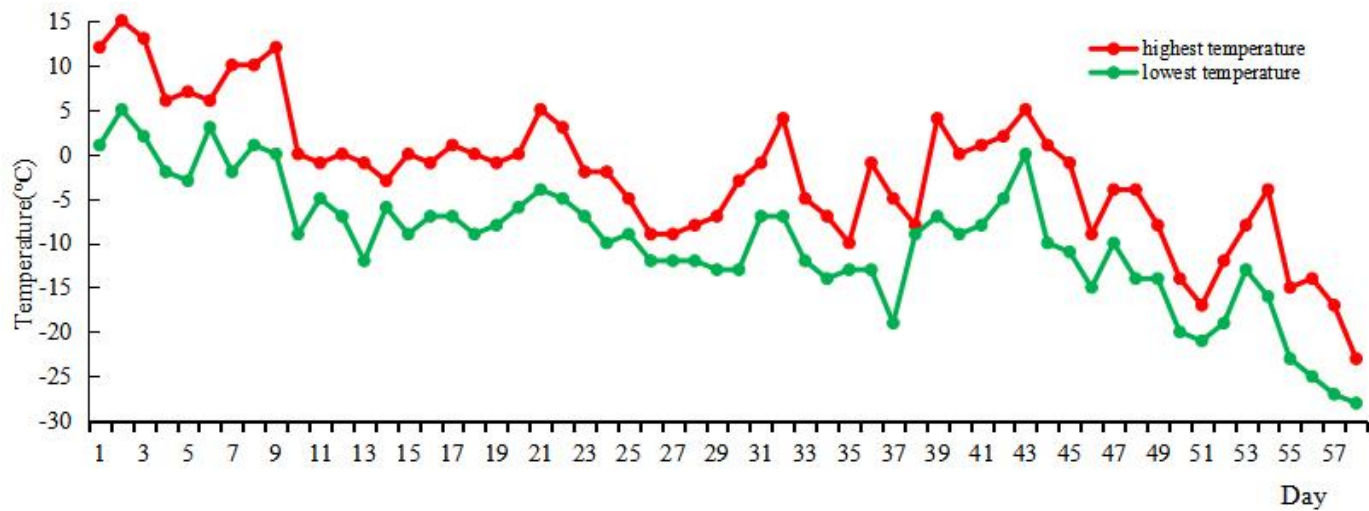

Fig.S1 Environmental temperature during the experiment

Supplement: Supplementary file 1 [file vetsci-13-00442-s001.zip › Fig.S/Fig.S1.pdf]

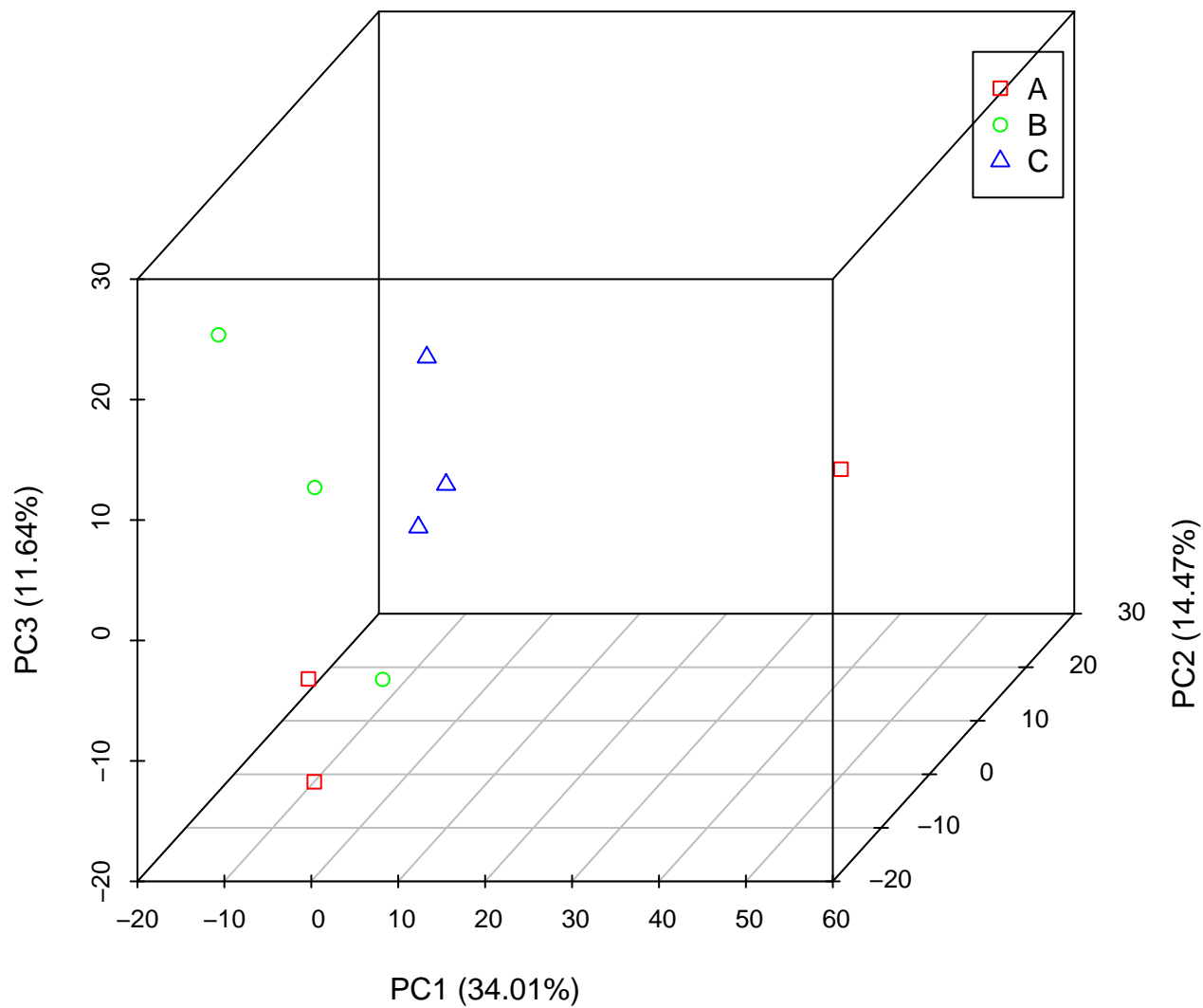

Fig.S2 The result of PCA

Supplement: Supplementary file 1 [file vetsci-13-00442-s001.zip › Fig.S/Fig.S2.pdf]
