# Supplementary material for: Gene Expression Profiling of Adipose Tissue in Enshi Black Pigs Subjected to Cold Stress
Source: Vet Sci. 2026 Apr 30;13(5):442. doi: 10.3390/vetsci13050442 (PMC13211509; doi:10.3390/vetsci13050442)
Supplement: Supplementary file 1 [file Fig.S4.pdf › Fig.S/Fig.S4.pdf]

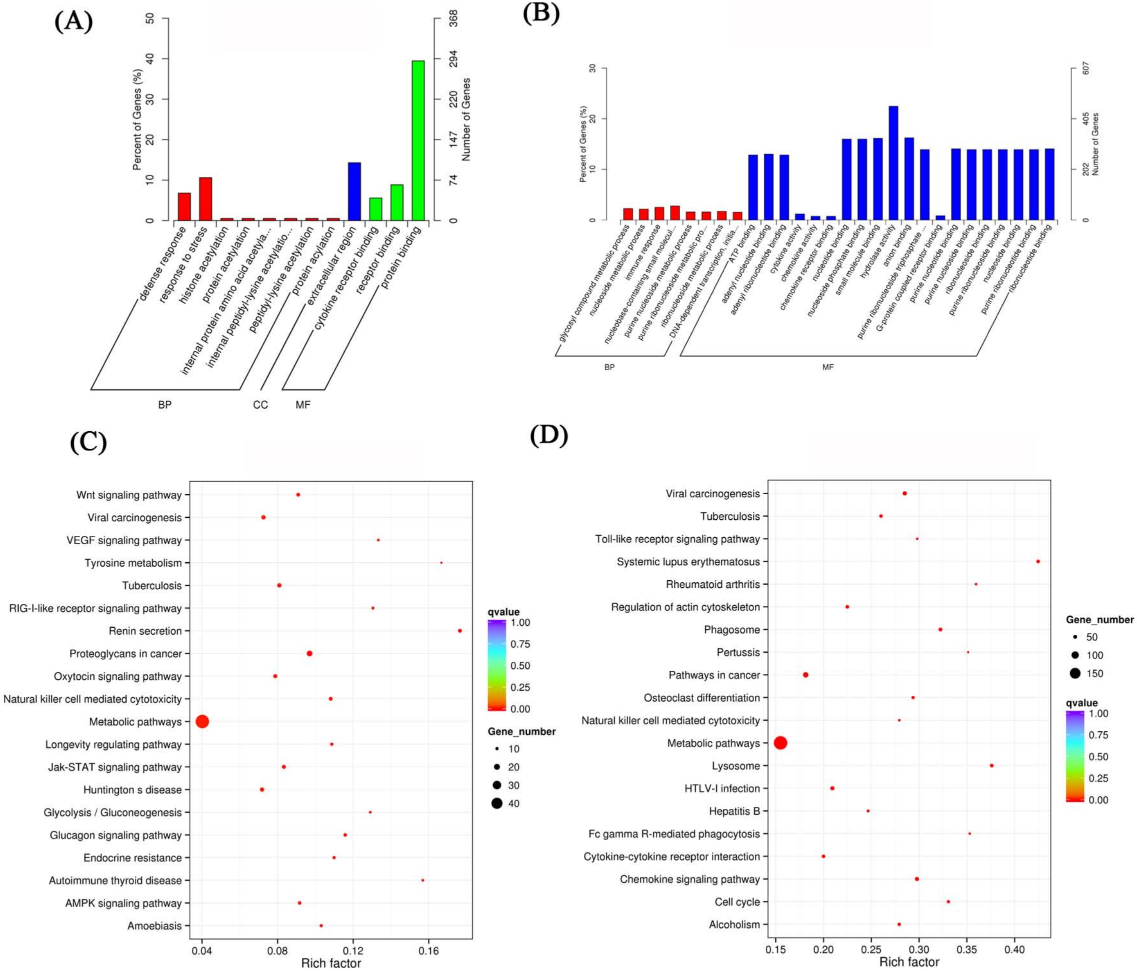

Fig. S4. Functional prediction of the DElncRNAs of group A. (A) GO analysis of DElncRNAs cis-targeted genes. (B) GO analysis of DElncRNAs trans-targeted genes. (C) Pathway analysis of DElncRNAs cis-targeted genes. (D) Pathway analysis of DElncRNAs trans-targeted genes.
